# Causes of death and clinical characteristics of 34 patients with Mucopolysaccharidosis II in Taiwan from 1995–2012

**DOI:** 10.1186/s13023-016-0471-6

**Published:** 2016-06-27

**Authors:** Hsiang-Yu Lin, Chih-Kuang Chuang, Yu-Hsiu Huang, Ru-Yi Tu, Fang-Ju Lin, Shio Jean Lin, Pao Chin Chiu, Dau-Ming Niu, Fuu-Jen Tsai, Wuh-Liang Hwu, Yin-Hsiu Chien, Ju-Li Lin, Yen-Yin Chou, Wen-Hui Tsai, Tung-Ming Chang, Shuan-Pei Lin

**Affiliations:** Department of Medicine, Mackay Medical College, New Taipei City, Taiwan; Department of Pediatrics, Mackay Memorial Hospital, No. 92, Sec. 2, Chung-Shan North Road, Taipei, 10449 Taiwan; Department of Medical Research, Mackay Memorial Hospital, Taipei, Taiwan; Mackay Junior College of Medicine, Nursing and Management, Taipei, Taiwan; Institute of Clinical Medicine, National Yang-Ming University, Taipei, Taiwan; Medical College, Fu-Jen Catholic University, Taipei, Taiwan; Institute of Biotechnology, National Taipei University of Technology, Taipei, Taiwan; Department of Pediatrics, Chi Mei Medical Center, Tainan, Taiwan; Department of Pediatrics, Kaohsiung Veterans General Hospital, Kaohsiung, Taiwan; Department of Pediatrics, Taipei Veterans General Hospital, Taipei, Taiwan; Department of Pediatrics, China Medical University Hospital, Taichung, Taiwan; Department of Pediatrics, National Taiwan University Hospital, Taipei, Taiwan; Division of Medical Genetics, Department of Pediatrics, Chang Gung Memorial Hospital at Linkou and Chang Gung University College of Medicine, Taoyuan, Taiwan; Department of Pediatrics, National Cheng Kung University Hospital, Tainan, Taiwan; Department of Pediatric Neurology, Changhua Christian Children’s Hospital, Changhua, Taiwan; Department of Infant and Child Care, National Taipei University of Nursing and Health Sciences, Taipei, Taiwan

**Keywords:** Cause of death, Hunter syndrome, Mortality, Mucopolysaccharidosis II, Taiwan

## Abstract

**Background:**

Mucopolysaccharidosis type II (MPS II) is an X-linked recessive, multisystemic lysosomal storage disorder caused by a deficiency of iduronate-2-sulfatase. MPS II has a variable age of onset and variable rate of progression. In Asian countries, there is a relatively higher incidence of MPS II compared to other types of MPS.

**Methods:**

A retrospective analysis was carried out of 34 Taiwanese MPS II patients who died between 1995 and 2012. The clinical characteristics, medical records, age at death, and cause of death were evaluated to better understand the natural progression of this disease.

**Results:**

The mean age at death of 31 of the patients with a severe form of the disease with significant cognitive impairment was 13.2 ± 3.2 years, compared with 22.6 ± 4.3 years in the three patients with a mild form of the disease without cognitive involvement (*n* = 2) or the intermediate form (*n* = 1) (*p* < 0.001). The mean ages at onset of symptoms and confirmed diagnosis were 2.5 ± 2.1 and 4.8 ± 3.1 years, respectively (*n* = 32). Respiratory failure was the leading cause of death (56 %), followed by cardiac failure (18 %), post-traumatic organ failure (3 %), and infection (sepsis) (3 %) (*n* = 27). Age at onset of symptoms was positively correlated with life expectancy (*p* < 0.01). Longevity gradually increased over time from 1995 to 2012 (*p* < 0.05).

**Conclusions:**

Respiratory failure and cardiac failure were the two major causes of death in these patients. The life expectancy of Taiwanese MPS II patients has improved in recent decade.

## Background

Mucopolysaccharidosis type II (MPS II; Hunter syndrome; OMIM +309900) is an X-linked recessive, multisystemic lysosomal storage disorder caused by deficient activity of iduronate-2-sulfatase (IDS), which catalyzes a sequential step in the catabolism of glycosaminoglycans (GAGs), heparan sulfate and dermatan sulfate. The accumulation of GAGs in the lysosomes of many organs and tissues causes progressive cellular dysfunction, alters the development of vital organs and the skeleton, and in severe cases leads to neurodegeneration and death by adolescence. MPS II has a variable age at onset and variable rate of progression. Patients with severe MPS II usually present between 2 and 4 years of age with coarse facial features, airway obstruction, cardiomyopathy, cardiac valve dysplasia, hepatosplenomegaly, joint stiffness, skeletal deformities, hernias, recurrent ear, nose, and throat infections, and progressive neurological involvement, leading to profound cognitive impairment and hyperactivity. Patients with the mild form of the disease are spared cognitive impairment, but still have other somatic problems [1–3].

Hematopoietic stem cell transplantation (HSCT) is currently the only treatment to prevent progressive neurodegenerative disease in a select group of MPS disorders, including MPS I, II, VI, and VII. However, its use is limited by a high risk of graft failure and transplantation-related morbidity and mortality [4–6]. Over the last few years, enzyme replacement therapy (ERT) with recombinant IDS (idursulfase; Elaprase, Shire Human Genetic Therapies, Cambridge, MA, USA) has been licensed in the United States and the European Union for the treatment of MPS II, and been shown to improve endurance, joint mobility, and lung function, and to potentially be beneficial for many patients with MPS II, especially if started early in the course of the disease [2, 7–9].

The incidence of MPS II differs among different populations, with reported rates ranging from 1.39 in 100,000 male live births in Northern Ireland [10] to 0.19 in 100,000 male live births in British Columbia [11]. Asian countries have a higher reported incidence of MPS II compared to other types of MPS, and in Taiwan the incidence of MPS II is approximately 2.05 in 100,000 male live births [12, 13]. A founder effect may account for the variations in the incidence in different ethnic populations.

Only a handful of reports have described the natural history and cause of death in patients with MPS II [14, 15], including the Hunter Outcome Survey (HOS) [14]. Although the enrolled cohort was large including 129 historical patients from 16 countries, only 3 % (*n* = 4) of those enrolled were Asian. Another related study was by Sohn et al. [15], who reported the natural history of 19 Korean patients with MPS II who died. The purpose of this study was to retrospectively collect and analyze data on the life expectancy and causes of death as recorded on the medical charts of Taiwanese MPS II patients who died between 1995 and 2012 to better understand the natural progression of this disease.

## Methods

### Study population

In order to acquire as much data as possible on all patients with MPS II in Taiwan who died from January 1995 to December 2012, the following sources were used: (1) Membership list of Taiwan MPS Society (patient support group); (2) Medical records from 8 medical centers in Taiwan, including Mackay Memorial Hospital, Taipei; National Cheng Kung University Hospital, Tainan; Kaohsiung Veterans General Hospital; National Taiwan University Hospital, Taipei; China Medical University Hospital, Taichung; Chi Mei Medical Center, Tainan; Taipei Veterans General Hospital; and Chang Gung Memorial Hospital, Taoyuan; (3) Laboratory records from the Department of Medical Research, Mackay Memorial Hospital, Taipei, Taiwan; and (4) Records from the Taiwan Foundation for Rare Disorders.

The diagnosis of all patients was confirmed by two-dimensional electrophoresis of urinary GAGs and a deficiency of IDS activity measured in peripheral leukocytes or fibroblasts [16, 17]. The clinical characteristics, medical records, age at death, and cause of death of these patients were retrospectively reviewed. The hospital’s ethics committee approved the study. All of the patients or their parents provided written informed consent.

### Data and statistical analysis

Descriptive statistics were performed, and the results are presented as mean ± standard deviation unless otherwise indicated. The relationships between life expectancy and year of death as well as age at onset of symptoms in these MPS II patients were evaluated using Pearson’s correlation coefficient (*r*), and testing for statistical significance (*p* < 0.05) was performed using Fisher’s *r-z* transformations. All statistical analyses were performed with SPSS version 11.5 (SPSS Inc., Chicago, Illinois, USA). Statistical significance was set at *p* < 0.05.

## Results

**Fig. 1:**
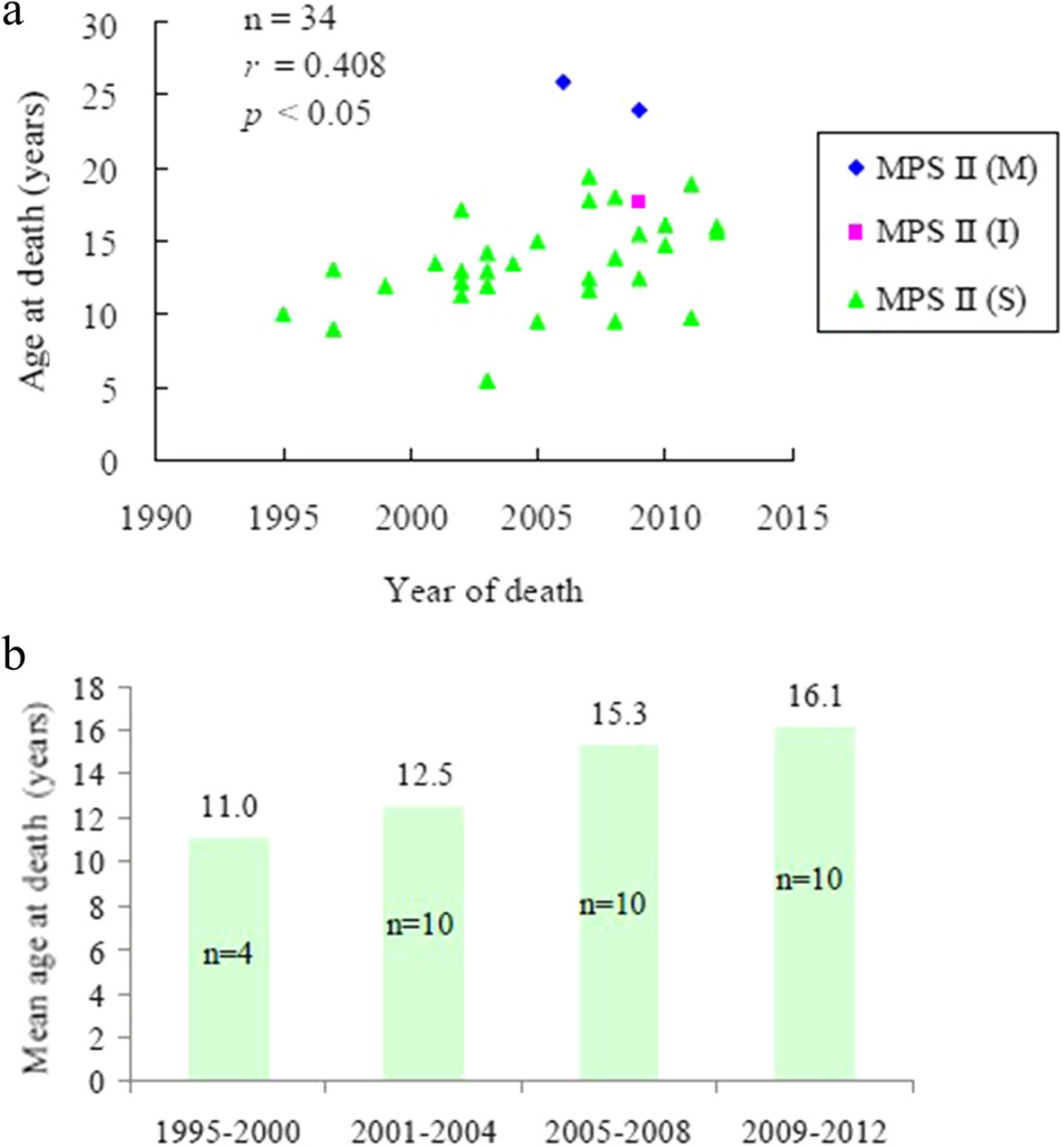
Life-span of Taiwanese MPS II patients who died between 1995 and 2012. **a** Age at death by individual patient. **b** Mean age at death over time. The life-span increased gradually over time (*p* < 0.05). MPS, mucopolysaccharidosis; (M), mild form; (I), intermediate form; (S), severe form

**Fig. 2:**
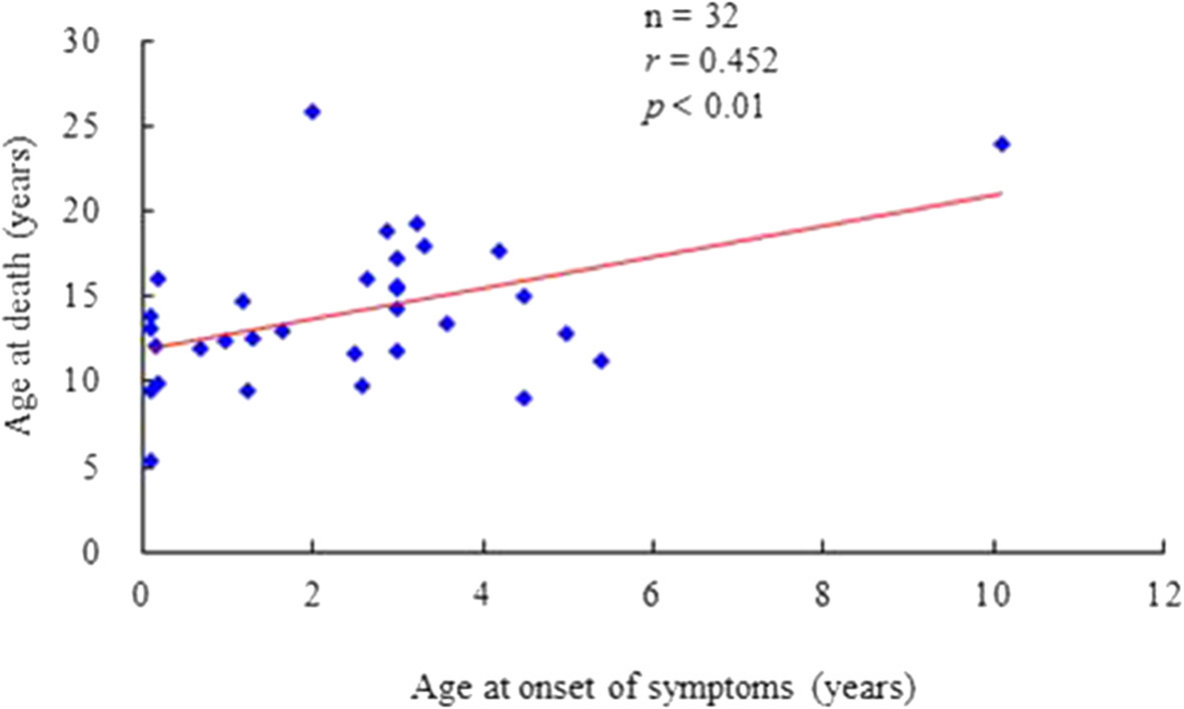
The relationship between age at onset of symptoms and life-span of Taiwanese MPS II patients who died between 1995 and 2012. Age at onset of symptoms was positively correlated with life expectancy (*p* < 0.01)

From January 1995 to December 2012, we identified 34 patients with MPS II who died. Among these patients, 31 had a severe form of disease with significant cognitive impairment, two had a mild form without cognitive involvement, and 1 had an intermediate form. In this cohort, two patients (No. 1 and No. 6) had received HSCT. Patient No. 1 received HSCT twice in 1999 and 2001, however, he died at the age of 5.4 years due to infection and sepsis. Patient No. 6 received HSCT in 1995 at 10 years of age, however he died at the same year due to cardiac failure. Only two patients (No. 28 and No. 33) had received ERT. Patient No. 28 received ERT for 1 year from September 2007 to September 2008, however he died in December 2009 at 17.7 years of age due to cardiac failure. Patient No. 33 received ERT for 1 month just before his death at the age of 24.1 years due to cardiac failure (Table 1). The mean age at death of all patients was 14.2 ± 4.2 years (median age 13.4 years), and the mean ages at onset of symptoms and confirmed diagnosis were 2.5 ± 2.1 and 4.8 ± 3.1 years, respectively (*n* = 32). The mean gestational age and birth weight were 39.2 ± 1.8 weeks and 3522 ± 581 grams, respectively (*n* = 24). The mean standard deviation score of birth weight was 0.56 ± 1.30 (*n* = 24). The primary cause of death was identified in 27 patients, while 7 patients (21 %) died without a definite cause being recorded. Respiratory failure was the leading cause (56 %) of death, followed by cardiac failure (18 %), post-traumatic organ failure (3 %), and infection (sepsis) (3 %) (Table 2). The mean age at death of the 31 patients with the severe form was 13.2 ± 3.2 years, compared with 22.6 ± 4.3 years in the three patients with the mild or intermediate forms (*p* < 0.001). Longevity also gradually increased over time from 1995 to 2012 (*p* < 0.05) (Fig. 1a). To further investigate this trend, we divided the year of death into four groups: 1995-2000, 2001-2004, 2005-2008, and 2009-2012. The mean ages at death were 11 years (1995-2000, *n* = 4), 12.5 years (2001-2004, *n* = 10), 15.3 years (2005-2008, *n* = 10), and 16.1 years (2009-2012, *n* = 10), respectively (Fig. 1b). The age at onset of symptoms was positively correlated with life expectancy (*p* < 0.01) (Fig. 2).

**Table 1:** Clinical characteristics of 34 Taiwanese MPS II patients who died between 1995 and 2012

No.	MPS type	Gender	Age at death (years)	Year at death	Age at onset of symptoms (years)	Age at diagnosis (years)	Gestational age (weeks)	Birth weight (grams)	Primary cause of death	Age at initial ERT (years)	Age at HSCT (years)
1	II (S)	M	5.4	2003	0.1	0.3	37	2800	Infection (sepsis)	–	1.1 and 3.1, respectively
2	II (S)	M	9.0	1997	4.5	7	NA	NA	Unknown	–	–
3	II (S)	M	9.5	2005	1.3	3.6	38	4700	Respiratory failure	–	–
4	II (S)	M	9.5	2008	0.1	1.2	37	2856	Respiratory failure	–	–
5	II (S)	M	9.8	2011	2.6	2.6	39	3450	Respiratory failure	–	–
6	II (S)	M	10.0	1995	0.2	9.3	41	3700	Cardiac failure	–	9.6
7	II (S)	M	11.3	2002	5.4	5.8	41	3200	Respiratory failure	–	–
8	II (S)	M	11.7	2007	2.5	3	40	3250	Respiratory failure	–	–
9	II (S)	M	11.9	1999	3	5.3	36	2800	Unknown	–	–
10	II (S)	M	11.9	2003	0.7	1.3	35	3150	Respiratory failure	–	–
11	II (S)	M	12.2	2002	0.2	5.1	39	3750	Respiratory failure	–	–
12	II (S)	M	12.4	2007	1	2.8	41	3900	Respiratory failure	–	–
13	II (S)	M	12.5	2009	1.3	1.3	NA	NA	Respiratory failure	–	–
14	II (S)	M	12.9	2002	5	6.7	38	4200	Unknown	–	–
15	II (S)	M	13.0	2003	1.7	5.0	40	3200	Respiratory failure	–	–
16	II (S)	M	13.1	1997	0.1	5	39	3870	Cardiac failure	–	–
17	II (S)	M	13.4	2001	NA	NA	NA	NA	Respiratory failure	–	–
18	II (S)	M	13.4	2004	3.6	5.5	40	3500	Respiratory failure	–	–
19	II (S)	M	13.9	2008	0.1	3.3	37	2400	Unknown	–	–
20	II (S)	M	14.3	2003	3	10.6	42	3695	Respiratory failure	–	–
21	II (S)	M	14.8	2010	1.2	1.5	40	3000	Cardiac failure	–	–
22	II (S)	M	15.1	2005	4.5	7.6	40	3000	Respiratory failure	–	–
23	II (S)	M	15.5	2009	3	3	NA	NA	Unknown	–	–
24	II (S)	M	15.6	2012	3	4	40	3550	Unknown	–	–
25	II (S)	M	16.1	2012	2.7	3.2	NA	NA	Respiratory failure	–	–
26	II (S)	M	16.1	2010	0.2	2	40	4150	Respiratory failure	–	–
27	II (S)	M	17.2	2002	3	12.2	NA	NA	Post-traumatic organ failure	–	–
28	II (I)	M	17.7	2009	4.2	4.3	40	3750	Cardiac failure	15.4	–
29	II (S)	M	17.8	2007	NA	NA	NA	NA	Unknown	–	–
30	II (S)	M	18.0	2008	3.3	3.3	41	4500	Respiratory failure	–	–
31	II (S)	M	18.9	2011	2.9	2.9	NA	NA	Respiratory failure	–	–
32	II (S)	M	19.4	2007	3.3	3.3	40	4160	Respiratory failure	–	–
33	II (M)	M	24.1	2009	10.1	10.1	NA	NA	Cardiac failure	23.9	–
34	II (M)	M	25.9	2006	2	10.8	NA	NA	Cardiac failure	–	–

*MPS* mucopolysaccharidosis, *ERT* enzyme replacement therapy, *HSCT* hematopoietic stem cell transplantation, (*S*) severe form, (*I*) intermediate form, (*M*) mild form, *NA* not available

**Table 2:** Clinical characteristics of the 34 Taiwanese patients with MPS II who died between 1995 and 2012

Clinical characteristics	Number (%)
Age at onset of symptoms (years) (*n* = 32)	2.5 ± 2.1
Age at diagnosis (years) (*n* = 32)	4.8 ± 3.1
Gestational age (weeks) (*n* = 24)	39.2 ± 1.8
Birth weight (gram) (*n* = 24)	3522 ± 581
Birth weight (SDS) (*n* = 24)	0.56 ± 1.30
Primary cause of death, *n* (%)	
Respiratory failure	19 (56 %)
Cardiac failure	6 (18 %)
Post-traumatic organ failure	1 (3 %)
Infection (sepsis)	1 (3 %)
Unknown	7 (21 %)
Age at death (years)	14.2 ± 4.2
Age at death, *n* (%)	
<5 years	0
≧5 to <10 years	5 (15 %)
≧10 to <15 years	16 (47 %)
≧15 to <20 years	11 (32 %)
≧20 years	2 (6 %)
Year of death, *n* (%)	
1995–2000	4 (12 %)
2001–2004	10 (29 %)
2005–2008	10 (29 %)
2009–2012	10 (29 %)
Phenotype, *n* (%)	
Severe	31 (91 %)
Intermediate	1 (3 %)
Mild	2 (6 %)
ERT, *n* (%)	
Yes	2 (6 %)
No	32 (94 %)
HSCT, *n* (%)	
Yes	2 (6 %)
No	32 (94 %)

*MPS* mucopolysaccharidosis, *SDS* standard deviation score, *ERT* enzyme replacement therapy, *HSCT* hematopoietic stem cell transplantation

## Discussion

Only a few studies have reported the survival and causes of death of patients with MPS II [14, 15], and to the best of our knowledge, this is the first multicenter study to analyze the life expectancy and causes of death of patients with MPS II in Taiwan. We found that the life expectancy of these patients has improved in recent decades. With the implementation of the National Health Insurance program in Taiwan in 1995, it is possible that this improvement may be due to referral of these patients to specialists and improvements in multidisciplinary care. We also found that the patients with cognitive impairment had a shorter life span than those without cognitive impairment, which is consistent with previous reports [14, 15].

The most severely affected patients with MPS II usually only survive until the second decade of life, however, less severely affected patients may survive until the fifth or sixth decades of life [2]. In a study by Jones et al. [14] on 129 patients from the HOS, the median age of death was 13.4 years, compared to 16.8 years in a study by Sohn et al. [15] on 19 Korean patients. The median age of death in the current study was similar at 13.4 years. Notably, all of the patients with the severe form of MPS II in this study died before 20 years of age, with the oldest age being 19.4 years.

For the patients with the mild form of MPS II without cognitive impairment, the median ages at death were 14.6 years (*n* = 62) and 25.1 years (*n* = 4) in the studies by Jones et al. [14] and Sohn et al. [15], respectively, which are similar to the current study (25 years; *n* = 2). Our previous cohort [12] showed that among 68 Taiwanese patients with MPS II diagnosed from 1984 to 2004, 49 (72 %) has the severe form with cognitive impairment, and 19 (28 %) had the mild form without cognitive impairment. According to Taiwanese governmental policy, ERT is only subsidized by the National Health Insurance program for MPS II patients who do not have neurological involvement. At the time of writing this study, 18 patients with the mild form of MPS II without neurological involvement are receiving ERT in Taiwan. Because most Taiwanese MPS II patients with the mild form are still alive, only two patients with the mild form (6 %) who died were enrolled in this study. Thus, the present study may not completely reflect the true survival rate of Taiwanese patients with the mild form of MPS II. In addition, since ERT for MPS II patients has been shown to substantially improve endurance, joint mobility, cardiac hypertrophy, and lung function [2, 7–9, 18], the length of survival and natural course of these patients may be significantly altered by ERT. Because only two patients in this study had received ERT (for 1 year and 1 month, respectively), the effects of ERT on mortality in these patients is not clear. Therefore, further multicenter studies with larger cohorts and a longer follow-up period are warranted.

HSCT is not indicated for patients with MPS II due to significant morbidity and mortality as well as no obvious efficacy shown in cognitive involvement [19, 20]. However, Tanaka et al. [6] performed a nationwide retrospective study in Japan on the efficacy of HSCT in 21 MPS II patients, and reported that HSCT was effective for brain or heart involvement when performed before signs of brain atrophy or valvular regurgitation. In the current study, two patients (No. 1 and No. 6) had received HSCT. Patient No. 1 received HSCT twice in 1999 and 2001, however he died at 5.4 years of age due to infection and sepsis. Patient No. 6 received HSCT in 1995 at 10 years of age, however he died the same year due to cardiac failure. The poor outcome of the latter patient may be due to the older age at the time of HSCT.

Airway problems are very common in patients with MPS, and airway-related morbidity and mortality is common, as reported in the HOS for MPS II. Cardiac dysfunction due to structural damage can also significantly increase the morbidity and mortality of affected patients [14, 21–23]. The leading cause of death in the HOS (*n* = 129) was airway problems (*n* = 59, 46 %), followed by cardiac problems (*n* = 20, 16 %) [14]. Sohn et al. [15] reported that the leading cause of death was pneumonia (*n* = 5, 26 %) among 19 patients with MPS II, with one patient dying due to myocardial infarction with coronary artery stenosis. In the current study, respiratory failure was the primary cause of death (*n* = 19, 56 %), followed by cardiac failure (*n* = 6, 18 %), which is consistent with the previous studies.

Różdżyńska-Świątkowska et al. [24] reported that many MPS patients are larger than the general population at the time of birth. Therefore, a high birth weight and/or large for gestational age may raise the suspicion of MPS and help to promptly identify the disease. In the present study, the mean gestational age and birth weight were 39.2 ± 1.8 weeks and 3522 ± 581 grams, respectively (*n* = 24) with a mean standard deviation score of birth weight of 0.56 ± 1.30 (*n* = 24), which is consistent with the previous studies.

Since MPS II is a rare, progressive and multisystemic disease, as well as insidious initial signs and symptoms in these patients, making an early diagnosis can be a challenge for first-line general medical practitioner. Sohn et al. [15] reported that the mean ages at onset of symptoms and at diagnosis in Korean MPS II patients were 3.3 years and 5.6 years, respectively (*n* = 75). The duration between these two time points is 2.3 years, which is consistent with our findings (2.3 years).

In the current study, the age at onset of symptoms was positively correlated with life expectancy (*p* < 0.01). It is well known that MPS II has a wide spectrum of disease severity, and that patients with the milder form may exhibit more insidious clinical signs and milder symptoms as well as a longer survival.

In this study, the life expectancy of the patients increased gradually over time from 1995 to 2012 (*p* < 0.05). With the implementation of the National Health Insurance program in Taiwan in 1995, it is possible that this improvement in life expectancy is due to referral of the patients to specialists and improvements in multidisciplinary care. Similarly, Jones et al. [14] reported that the median age at death in the HOS was significantly lower in the patients who died in or before 1985 compared with those who died after 1985 (11.3 versus 14.1 years, *p* < 0.001). Sohn et al. [15] also reported that the patients who died after 2005 had a better survival than those who died before 2005 (19.4 versus 11.4 years, *p* < 0.05). This may reflect improvements in an early diagnosis, medical care, and appropriate treatment for patients over the past two decades.

### Limitations

As a retrospective and multicenter study, some medical records were missing and not available at the time of this study. In addition, the small sample size in this cohort reflects the rare nature of this genetic disorder, and the degree of disease severity was quite variable. Therefore, studies with a larger cohort with a longer follow-up period are warranted.

## Conclusion

Respiratory failure and cardiac failure were the two major causes of death for the patients with MPS II in this study. The life expectancy of Taiwanese MPS II patients has improved in recent decades, possibly due to referral of patients to specialists and improvements in multidisciplinary care. These findings could serve as baseline data for the analysis of the long-term effects of ERT and HSCT on patients with MPS II, and to develop quality of care strategies.

## Abbreviations

ERT, enzyme replacement therapy; GAGs, glycosaminoglycans; HOS, Hunter Outcome Survey; HSCT, hematopoietic stem cell transplantation; IDS, iduronate-2-sulfatase; MPS II, mucopolysaccharidosis type II; MPS, mucopolysaccharidosis
